# Exogenous spermine pretreatment confers tolerance to combined high-temperature and drought stress *in vitro* in trifoliate orange seedlings via modulation of antioxidative capacity and expression of stress-related genes

**DOI:** 10.1080/13102818.2014.909152

**Published:** 2014-07-08

**Authors:** Xing-Zheng Fu, Fei Xing, Nan-Qi Wang, Liang-Zhi Peng, Chang-Pin Chun, Li Cao, Li-Li Ling, Cai-Lun Jiang

**Affiliations:** ^a^ Citrus Research Institute, Southwest University, Chongqing400712, P.R. China; ^b^ National Citrus Engineering Research Center, Chongqing400712, P.R. China; ^c^ College of Horticulture and Landscape, Southwest University, Chongqing400715, P.R. China

**Keywords:** citrus, polyamine, high-temperature and drought stresses, reactive oxygen species, antioxidant enzymes

## Abstract

Spermine (Spm) is thought to play an important role in drought or high-temperature (HT) tolerance. However, it is not clear whether Spm confers similar resistance in the presence of both drought and HT, which often occur simultaneously. In the present study, the trifoliate orange (*Poncirus trifoliata* (L.) Raf.) seedlings were pretreated with 1 mmol L^−1^ Spm to evaluate their tolerance to combined drought and HT (45 ºC) stress. Spm-pretreated seedlings showed less leaf wilting, less water loss and less electrolyte leakage than control leaves not treated with Spm within 180 min of treatment. Histochemical staining with diaminobenzidine and nitro blue tetrazolium showed that Spm-pretreated seedlings accumulated less hydrogen peroxide and superoxide than those of control plants 60, 120 and 180 min after treatment when exposed to both drought and HT (45 ºC). However, superoxide dismutase, peroxidase and catalase were significantly more active in Spm-pretreated seedlings than in control seedlings. In addition, Spm-pretreated seedlings showed significantly higher expression of heat shock proteins, abscisic acid (ABA)-responsive element binding factor and 9-cis-epoxycarotenoid dioxygenase 3 than controls either before (0 min) or after (60, 120 and 180 min) combined drought and HT treatment. All of these data suggest that exogenous Spm pretreatment confers tolerance to simultaneously occurring drought and HT stresses. Spm may influence this by activating antioxidant enzymes, increasing the effectiveness of scavenging of reactive oxygen species. It may also increase the expression levels of stress-related genes that protect trifoliate orange seedlings from stress damage.

## Introduction

Drought and high temperature (HT) are the major environmental stresses restricting plant growth and development. In citrus production, these two stresses occur frequently, often simultaneously. China's citrus production areas often face simultaneously occurring seasonal drought and HT. In the summer, drought can accompany air temperatures as high as 40 ºC, lasting about one month. Combined drought and HT stress can lead to defoliation and shoot withering, cause the fruit to crack and drop and even kill the citrus trees. Abundant water resources and a lot of labour are being invested in the control of drought and HT stresses in citrus industry every year. As global climate change continues, drought and HT may occur more often and more unpredictably. It is therefore necessary to develop suitable strategies that can alleviate the effects of these two stresses.

Drought and HT stresses can result in water loss in plant cells; there is overaccumulation of reactive oxygen species (ROS) such as hydrogen peroxide (H_2_O_2_) and superoxide (O_2_^−^), leading to membrane damage and lipid peroxidation.[1] To survive under these adverse environmental conditions, plants have developed a series of response mechanisms. The main response mechanisms involve autologous physiological, biochemical and molecular regulation.[2,3] They include the activation of a large number of stress-related genes, such as mitogen-activated protein kinases, calcium-dependent protein kinases and ABA-responsive elements (ABREs).[2,3] They also involve induction of the synthesis of various functional proteins, sugars, sugar alcohols, amino-acids and amines.[4,5] These proteins and compounds act as osmoprotectants, antioxidases and ROS scavengers, allowing re-establishment of plant homeostasis.[4] Among these compounds, polyamines are the most important of the protectants that accumulate under drought or HT stress conditions.[6,7]

Polyamines, mainly diamine putrescine (Put), triamine spermidine (Spd) and tetraamine spermine (Spm), are low-molecular-weight aliphatic polycations. They are ubiquitously distributed in all living organisms. The cationic properties of polyamines allow them to interact with various macromolecules, such as nucleic acids, proteins and phospholipids.[8] Polyamines are involved in many physiological processes, such as cell division, growth and development, and respond to stress tolerance to various environmental factors.[7–9] So far, a great deal of evidence has indicated that polyamines play important roles in drought and HT stress responses. Previous studies have shown that polyamine titers increase under drought or HT stress conditions in various plants.[6,7,9,10] The use of exogenous polyamines was found to improve drought or HT tolerance. Foliar sprays of 10 μmol L^−1^ Put, Spd or Spm improve the drought tolerance of rice seedlings.[11] Pretreatment with three polyamines also enhances the growth of mung beans and soybeans under HT stress conditions.[12,13] Yamaguchi et al. [14] found that Spm pretreatment could reverse the drought hypersensitive phenotype of an *Arabidopsis acl5*/*spms* mutant otherwise incapable of producing Spm. Overexpression of polyamine biosynthetic genes, such as arginine decarboxylase (ADC), Spd synthase (SPDS) and S-adenosylmethionine decarboxylase (SAMDC), confers drought or HT tolerance upon many plants, including rice, *Arabidopsis*, tomatoes and tobacco.[15–19]

This shows that increases in polyamine levels induced by exogenous application or accelerant biosynthesis may be an important approach to improve the drought or HT tolerance of plants. However, most of the evidence supporting this idea was obtained from studies that involved annual plants. There is very little information about perennial plants, especially citrus plants. Moreover, previous studies have analysed the role of polyamines in plants affected by a single type of stress at a given time. It is not clear whether polyamines can confer similar resistance in the presence of multiple and simultaneous stresses. To answer this question and to provide some theoretical foundation for overcoming combined drought and HT stress in citrus production by utilization of polyamines in the future, the effects of exogenous application of Spm *in vitro* in trifoliate orange seedlings subjected to simultaneous drought and HT stresses were evaluated in the present study.

## Materials and methods

### Plant materials and treatments

Trifoliate orange (*Poncirus trifoliata* (L.) Raf.) seedlings were used as experimental materials. The seed coats of trifoliate orange seeds were removed, and the seeds were placed on a salver covered with wet gauze (to maintain humidity) and germinated at 28 ºC in the dark for 15 days. Germinated seedlings were sorted by size and groups of seedlings of similar size were transferred to 7-L plastic containers containing a nutrient solution of the following composition: 4 mmol L^−1^ Ca(NO_3_)_2_, 6 mmol L^−1^ KNO_3_, 1 mmol L^−1^ NH_4_H_2_PO_4_, 2 mmol L^−1^ MgSO_4_, 46 μmol L^−1^ H_3_BO_3_, 6 μmol L^−1^ MnCl_2_, 0.7 μmol L^−1^ ZnSO_4_, 0.3 μmol L^−1^ CuSO_4_, 1 μmol L^−1^ H_2_MoO_4_ and 50 μmol L^−1^ Fe-EDTA. Seedlings were cultured in a growth chamber at 25 ºC with a 16-h light (50 μmol m^−2^ s^−1^)/8-h dark regime. During culture, the nutrient solution was aerated for 30 min at 2-h intervals and replaced every week. The pH was adjusted daily to 6.0 with 1 mol L^−1^ HCl.

Seedlings of similar size were selected after three months of culture in the nutrient solution. These were transferred to distilled water supplemented with 1 mmol L^−1^ Spm for 30-h pretreatment. Seedlings cultured in distilled water were used as controls. After pretreatment, both the Spm-pretreated and control seedlings were removed from the distilled water and surface-dried on filter paper. The seedlings were then placed in a growth chamber at 45 ºC for both HT and dehydration (drought) treatment. The total treatment time was 180 min, and the changes in seedling phenotype were recorded. Seedlings were photographed.

### Measurement of relative water loss and relative electrolyte leakage

To analyse the relative water loss (RWL), the fresh weight of the Spm-pretreated and control seedlings was measured at 20-min intervals beginning with HT and drought treatment. The initial fresh weight (0 min) was recorded as *M*_0_, and the fresh weight at other points in time (*x*) was recorded as *M_x_*. RWL was calculated at each point in time, using the following formula: RWL = (*M*_0_ − *M_x_*)/*M*_0_ × 100%. Relative electrolyte leakage (REL) was measured at 0, 30, 90, 120, 150 and 180 min at 45 ºC under drought conditions, as described by Shi et al.[20]

### *In situ* histochemical detection of H_2_O_2_ and O_2_^−^

*In situ* accumulation of H_2_O_2_ and O_2_^−^ was detected at 0, 60, 120 and 180 min of HT and dehydration treatment by histochemical staining with 3,3′-diaminobenzidine (DAB) and nitro blue tetrazolium (NBT), respectively. The protocol was conducted as described previously.[20]

### Analysis of superoxide dismutase, peroxidase and catalase activity

The activity levels of superoxide dismutase (SOD), peroxidase (POD) and catalase (CAT) were quantified at 0, 60, 120 and 180 min in plants subjected to HT and dehydration treatment, using detection kits in accordance with the manufacturer's instructions (Nanjing Jiancheng Bioengineering Institute, China).

### Expression analysis of stress-related genes via quantitative real-time polymerase chain reaction

Quantitative real-time polymerase chain reaction (qRT-PCR) was used to analyse the expression levels of stress-related genes, including heat shock protein 70 (*Hsp70*), *Hsp90*, *Hsp100*, ABRE binding factor (*ABF*) and 9-*cis*-epoxycarotenoid dioxygenase 3 (*NCED3*) in Spm-pretreated and control seedlings before and after simultaneous drought and HT treatment. For this purpose, total RNA was isolated from the Spm-pretreated and control samples, using the TRIZOL reagent (Invitrogen, Carlsbad, CA, USA). One microgram of the total RNA was used for cDNA synthesis using the ReverTra Ace-α-™ kit (Toyobo, Japan) following the manufacturer's instructions. The *Hsp90* (JQ281798.1), *ABF* (HM171703.1) and *NCED3* (DQ309342.1) genes of citrus trees have known sequences in the NCBI database, and the sequences of *Hsp70* and *Hsp100* in citrus were obtained by blasting the sweet orange (*Citrus sinensis*) genome,[21] using the relevant *Arabidopsis* genes as query sequence. The specific primers of these genes were designed using the online primer-blast program in the National Center of Biotechnology Information (NCBI) website. The PCR amplification was performed using the Bio-Rad iCycler iQ Real-Time PCR detection system and the SYBR^®^ *Premix Ex Taq*™ dye (TaKaRa, Tokyo, Japan) with the following programme: 95 ºC denaturation for 30 s, then 40 cycles of denaturation at 95 ºC for 5 s, annealing at 58 ºC for 20 s and elongation at 72 ºC for 20 s. Each sample was amplified in quadruplicate.

### Statistical analysis

The data presented herein are mean values of three independent replicates, shown here as the mean ± SE. The data were analysed using Statistical Analysis System (SAS) statistical software (Version 8.0, SAS Institution, NC, U.S). Analysis of variance was used to evaluate the statistical differences based on Fisher's Least Significant Difference (LSD) test, at levels of significance of *P* < 0.05 (*), *P* < 0.01 (**) and *P* < 0.001 (***).

## Results and discussion

### Spm pretreatment and the tolerance of trifoliate orange to combined HT and drought stress

**Figure 1.**
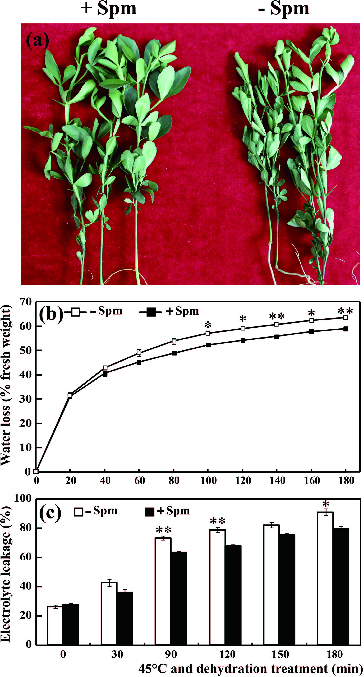
The phenotype (a), relative water loss (b) and electrolyte leakage (c) of Spm-pretreated (+Spm) and control (−Spm) trifoliate orange seedlings at 45 ºC under dehydration treatment conditions (* for *P* < 0.05, ** for *P* < 0.01).

The leaves of trifoliate orange seedlings not treated with Spm (control) wilted after 180 min of exposure to 45 ºC temperature and dehydration treatment (Figure 1(a)). However, only a few leaves of the Spm-pretreated seedlings showed even slight wilting (Figure 1(a)). Although the RWL and REL of both Spm-pretreated and control seedlings increased over time, the RWL and REL of Spm-pretreated seedlings remained significantly lower than those of controls at all points in time (Figure 1(b) and 1(c)). These results are consistent with those recorded for red tangerines pretreated with Spm and subjected to drought stress alone.[20]

The lower RWL of Spm-pretreated seedlings suggests that water evaporation may be slower under HT and drought conditions. This is further confirmed by the fact that exogenous application of polyamines induces stomatal closure in peanuts,[22,23] *Arabidopsis*,[14] and red tangerines.[20] In this way, it can be speculated that Spm pretreatment in trifoliate orange seedlings may promote the closure of stomata, reducing water loss, RWL and decreasing leaf wilting under combined stress conditions. REL is an indicator of cell membrane damage, which was also significantly less severe in Spm-pretreated samples than in controls. This suggests that Spm pretreatment may reduce the damage and lipid peroxidation of the cell membrane in trifoliate orange plants under combined HT and drought stress conditions.[6,8] Taken together, these results suggested that Spm pretreatment confers tolerance to combined HT and drought stress in trifoliate orange plants. This provides new proof that Spm plays important roles in the plant response to combined stresses. It also implies that Spm could possibly be used to overcome combined drought and HT stresses in citrus production. To provide more theoretical foundations and relevant information the mechanisms underlying the Spm conferred stress tolerance were further studied.

### Spm pretreatment and accumulation of ROS during combined HT and drought stress conditions

**Figure 2.**
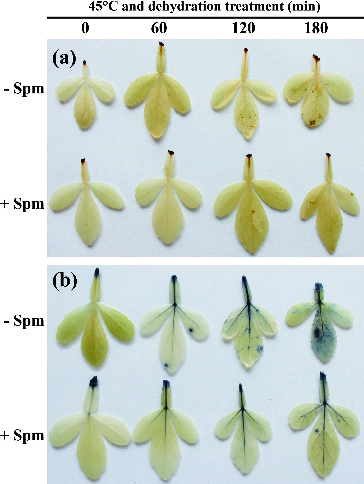
Accumulation of H_2_O_2_ (a) and O_2_^−^ (b) in Spm-pretreated (+Spm) and control (−Spm) trifoliate orange seedlings.

As mentioned above, over-accumulation of ROS is a common response to abiotic stress. It can lead to membrane damage and lipid peroxidation. To determine whether Spm pretreatment could reduce the accumulation of ROS, two main representatives of ROS, H_2_O_2_ and O_2_^−^, were detected using histochemical staining with DAB and NBT, respectively. The depth, area and number of brown and blue spots here represent the levels of H_2_O_2_ and O_2_^−^ in leaves, respectively. As shown in Figure 2(a), the number of brown spots gradually increased in control leaves as treatment continued. The brown spots became obvious 120 and 180 min after the beginning of HT and dehydration treatment. However, this symptom was not notable in Spm-pretreated leaves even after 180 min of stress treatment. Similar results were observed for NBT staining. There were significantly fewer blue spots in Spm-pretreated leaves than in control leaves, especially at 120 and 180 min of treatment (Figure 2(b)). These results suggested that under combined HT and drought conditions, Spm-pretreated seedlings accumulated significantly less H_2_O_2_ and O_2_^−^ than controls. This was closely consistent with the lower REL detected in these samples. The reduced accumulation of ROS after Spm pretreatment may be explained by the roles of polyamines in ROS scavenging described in previous reports. Polyamines can scavenge ROS directly or inhibit the production of ROS indirectly *via* competing with metal ions necessary for ROS formation, such as Fe^2+^ and Cu^2+^.[24–26] Alternatively, the activities of ROS-scavenging enzymes, such as CAT, POD and SOD, can be improved by polyamines. Relevant results have been reported in several plants.[11,20,27]

### Spm pretreatment and the activity of antioxidant enzymes under combined HT and drought stress conditions

**Figure 3.**
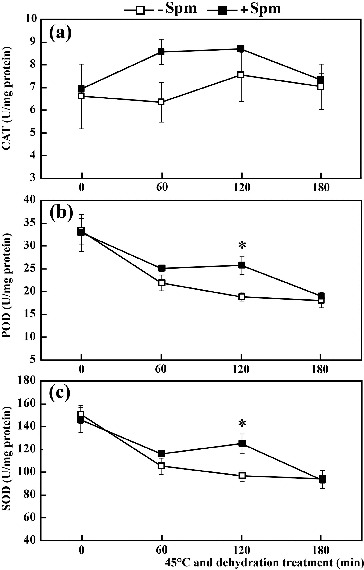
Activity of CAT (a), POD (b) and SOD (c) in Spm-pretreated (+Spm) and control (−Spm) trifoliate orange seedlings at 45 ºC under dehydration treatment conditions (* for *P* < 0.05).

The results given above indicated that Spm pretreatment reduced the accumulation of ROS under combined stress conditions. To determine whether Spm pretreatment could enhance the activity of antioxidant ROS scavenging enzymes, the activity of the three main antioxidant enzymes (CAT, POD and SOD) was quantified. Under dehydration conditions at 45 ºC, CAT activity first increased and then decreased, peaking at 120 min after treatment (Figure 3(a)). However, the activity of POD and SOD decreased continuously from 0 to 180 min, with lowest values appearing 180 min after treatment (Figure 3(b) and 3(c)). In Spm-pretreated seedlings, the activity levels of CAT, POD and SOD remained consistently higher than in controls, with one exception. No differences were observed in enzyme activity at either 0 or 180 min. POD and SOD activity remained statistically significantly different between Spm-pretreated seedlings and untreated controls at 120 min of combined stress treatment. These results indicate that Spm pretreatment can increase the activity of antioxidant enzymes under combined HT and drought stress conditions.

### Spm pretreatment and levels of expression of stress-related genes under combined HT and drought stress conditions

**Figure 4.**
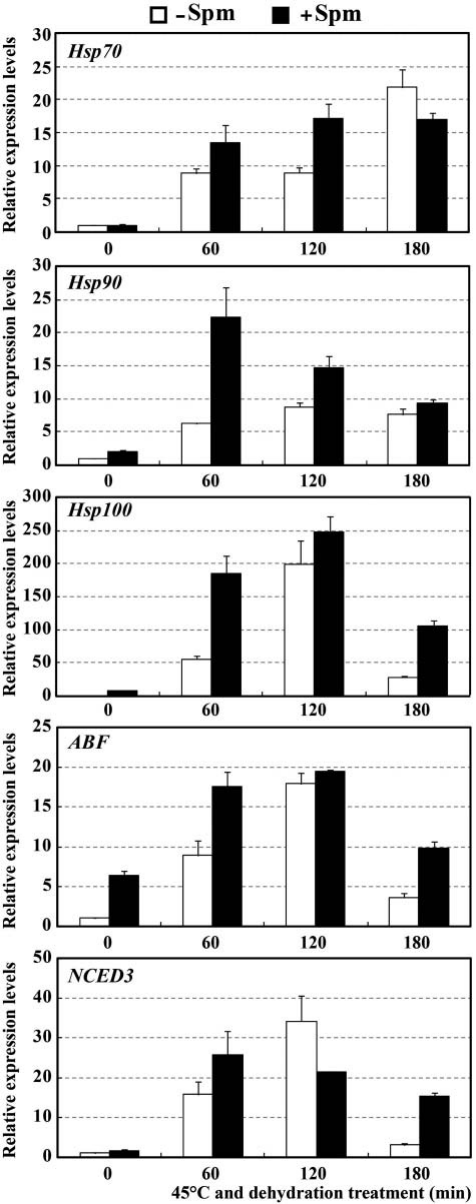
Expression levels of *Hsp70*, *Hsp90*, *Hsp100*, *ABF* and *NCED3* genes in Spm-pretreated (+Spm) and control (−Spm) trifoliate orange seedlings at 45 ºC under dehydration treatment conditions.

It has been proposed that polyamines, Spd and Spm in particular, may serve not only as important functional protectants, but also as signalling molecules in the induction of gene expression and enhancement of the DNA-binding activity of transcription factors under biotic and abiotic stress conditions.[28–30] For this reason, the transcription levels of several stress-related genes were investigated in Spm-pretreated seedlings and Spm-unpretreated controls. As shown in Figure 4, in both Spm-pretreated and untreated control seedlings, all tested genes, *Hsp70*, *Hsp90*, *Hsp100*, *ABF* and *NCED3*, showed significantly higher expression levels at 60, 120 and 180 min of HT and drought treatment than that at 0 min. The expression levels of these genes in Spm-pretreated seedlings were markedly higher than those in controls at all points in time, with two exceptions, the *Hsp70* gene at 180 min and the *NCED3* gene at 120 min. These data suggest that although these five stress-related genes were induced in both Spm-pretreated and control seedlings under combined stress conditions, treated plants showed more intense induction, even to the point where they seemed free of stress.

Hsp70, Hsp90 and Hsp100 are the primary Hsps responsible for thermotolerance. These Hsps act as molecular chaperones, interacting with other proteins to prevent stress-induced protein denaturation and aggregation.[31,32] The induction of *Hsp70*, *Hsp90* and *Hsp100* genes observed in the present study upon exogenous application of Spm was found to be closely consistent with the results of several previous reports.[31,33] This suggested that Spm might cause plants to synthesize more abundant Hsps to protect them from stress damage. ABFs are basic region/leucine zipper (bZIP) class transcription factors involved in the transcriptional regulation of ABA- and/or stress-responsive genes *via* interaction with ABRE *cis*-elements in their promoters.[29,34] Numerous studies have shown that ABFs play important roles in drought tolerance.[29,34] NCED is a key rate-limiting enzyme in ABA biosynthesis, and overexpression of this gene has been observed to cause overproduction of endogenous ABA, increase of stomatal closure and enhancement of drought tolerance in several species.[35–37] Stronger induction of *ABF* and *NCED3* genes in Spm-pretreated seedlings than in controls indicates that Spm-pretreated seedlings had more powerful transcriptional regulation and efficient ABA biosynthesis, which is required for stress tolerance. Based on our findings and previous reports, it can be speculated that Spm may induce the expression of stress-related genes as a signalling molecule under either normal or stress conditions. It is also the molecular mechanism underlying the stress tolerance conferred by exogenously applied Spm.

## Conclusions

The present study showed that Spm pretreatment can enhance the tolerance of trifoliate orange plants to combined HT and drought. The reduced wilting of leaves and lower concentrations of RWL and REL observed here support this conclusion. The exogenous application of Spm probably allows the plants to maintain higher levels of antioxidant enzymes, stronger scavenging of ROS and higher expression of stress-related genes under combined stress conditions. This may reduce the damage to cell membrane, lower lipid peroxidation and prevent stress-induced protein denaturation and aggregation to protect the trifoliate orange seedlings from stress damage, as seen in the present study. The current work provides new evidence supporting that polyamines confer tolerance to multiple and simultaneous stresses. It also describes a novel method of enhancing simultaneous drought and HT stress tolerance in a perennial fruit crops.
